# Quantifying the Landscape of Decision Making From Spiking Neural Networks

**DOI:** 10.3389/fncom.2021.740601

**Published:** 2021-10-28

**Authors:** Leijun Ye, Chunhe Li

**Affiliations:** ^1^Institute of Science and Technology for Brain-Inspired Intelligence, Fudan University, Shanghai, China; ^2^Shanghai Center for Mathematical Sciences, Fudan University, Shanghai, China; ^3^School of Mathematical Sciences, Fudan University, Shanghai, China

**Keywords:** decision making, neural network, attractor, energy landscape, kinetic path

## Abstract

The decision making function is governed by the complex coupled neural circuit in the brain. The underlying energy landscape provides a global picture for the dynamics of the neural decision making system and has been described extensively in the literature, but often as illustrations. In this work, we explicitly quantified the landscape for perceptual decision making based on biophysically-realistic cortical network with spiking neurons to mimic a two-alternative visual motion discrimination task. Under certain parameter regions, the underlying landscape displays bistable or tristable attractor states, which quantify the transition dynamics between different decision states. We identified two intermediate states: the spontaneous state which increases the plasticity and robustness of changes of minds and the “double-up” state which facilitates the state transitions. The irreversibility of the bistable and tristable switches due to the probabilistic curl flux demonstrates the inherent non-equilibrium characteristics of the neural decision system. The results of global stability of decision-making quantified by barrier height inferred from landscape topography and mean first passage time are in line with experimental observations. These results advance our understanding of the stochastic and dynamical transition mechanism of decision-making function, and the landscape and kinetic path approach can be applied to other cognitive function related problems (such as working memory) in brain networks.

## 1. Introduction

The brain operates as a complex non-linear dynamical system, performing various physiological or cognitive functions. The computational ability emerges when a collection of neurons richly interact with each other via excitation or inhibition. Decision making is a cognitive process in terms of choosing a particular action or opinion among a set of alternatives, governing the behavioral flexibility and pervading all aspects of our life (Lee, 2013). Decision making process for sensory stimuli, such as the interpretation of an ambiguous image (Sterzer et al., 2009; Wang et al., 2013) or the discrimination of motion direction of random dots (Shadlen and Newsome, 2001; Roitman and Shadlen, 2002; Churchland et al., 2008; Lin et al., 2020), is closely associated with lateral intraparietal cortex (area LIP), which receives the inputs from sensory cortex and guides the motor output.

Decision making functions have been successfully described by the attractor network-based framework (Wang, 2002; Wong and Wang, 2006; Wong et al., 2007; Deco et al., 2013; You and Wang, 2013; Murray et al., 2017), which is characterized by its ability to account for the persistent activity observed broadly across many decision-related neurons. In the attractor network model, the persistent activity of the neural populations can be sustained, i.e., the system settles in the decided attractor state, even after the withdrawal of the external stimulus (Wang, 2002; Wong and Wang, 2006).

A concept closely related to attractor state is multistability, which refers to the coexistence of multiple steady states and exists in both a single neuron and neuronal populations in the brain, evidenced by a number of theoretical and experimental studies (Kelso, 2012). For example, two patterns of neuronal oscillations in cortical neurons, slow oscillations vs. tonic and irregular firing, correspond to two different cortical states, slow-wave sleep vs. wakefulness (Shilnikov et al., 2005; Fröhlich and Bazhenov, 2006; Destexhe et al., 2007; Sanchez-Vives et al., 2017). Perceptual multistability in visual and auditory systems is closely related to the coordination of a diversity of behaviors (Kondo et al., 2017). Furthermore, the brain is essentially noisy, originating from stochastic external inputs and highly irregular spiking activity within cortical circuits (Wang, 2008; Braun and Mattia, 2010). In multistable system, the noise induces alternations between distinct attractors in an irregular manner. For example, during extended gazing of a multistable image, inherent brain noise is responsible for inducing spontaneously switches between coexisting perceptual states (Braun and Mattia, 2010). The alternative switching between coexisting mental states for thinking process is also triggered by neuronal brain noise, either spontaneously or initiated by external stimulation (Kelso, 2012). Despite many advances on attractor networks and multistability in neural systems, the stochastic transition dynamics and global stability for decision making in neural networks have yet to be fully clarified.

For non-equilibrium dissipative dynamical systems such as biological neural circuits, the non-equilibrium potential (NEP) can be defined to facilitate the quantification of the global stability (Ludwig, 1975; Graham, 1987; Ao, 2004). Hopfield pioneeringly proposed the qualitative concept of "energy function" to explore the computational properties of neural circuits, such as the associative memory (Hopfield, 1984; Hopfield and Tank, 1986; Tank and Hopfield, 1987). The underlying energy landscape provides a global and quantitative picture of system dynamics which potentially can be used to study the stochastic transition dynamics of neural networks and has been described extensively in the literature, but mostly as illustrations (Walczak et al., 2005; Wong and Wang, 2006; Moreno-Bote et al., 2007; Braun and Mattia, 2010; Fung et al., 2010; Rolls, 2010; Zhang and Wolynes, 2014). Recently, some efforts have been devoted to quantifying the energy landscape from mathematical models. For the neural oscillations related to cognitive process, for example, the rapid-eye movement sleep cycle, the landscape shows a closed-ring attractor topography (Yan et al., 2013). The energy landscape theory also uncovers the essential stability-flexibility-energy trade-off in working memory and the speed-accuracy trade-off in decision making (Yan et al., 2016; Yan and Wang, 2020). However, these attempts are based on the simplified biophysical model rather than more realistic spiking neural network model, which may not fully capture some key aspects of the corresponding cognitive function (Wong and Wang, 2006). Therefore, how to quantify the energy landscape of cognitive systems based on a spiking neural network is still a challenging problem. Besides, the non-equilibrium landscape and flux framework has been applied to explore the cell fate decision-making based on gene regulatory networks (Wang et al., 2008; Li and Wang, 2013a,b, 2014a; Lv et al., 2015; Ge and Qian, 2016; Ye et al., 2021). So in this work, we focus on quantifying the energy landscape from a more plausible spiking cell-based neural network model to study the underlying stochastic dynamics mechanism of perceptual decision making in the brain.

In this work, we aim at quantifying the attractor landscape and further the stochastic dynamics and global stability of the decision making function from the spiking neural network model. The model we employed here was firstly introduced in Wang (2002), characterized by the winner-take-all competition mechanism for the binary decision. Depending on the parameter choice, the underlying neural circuit displays up to four stable attractors, which characterizes decision states, spontaneous state and intermediate states, individually. The irreversibility of the kinetic transition paths between attractors is due to the probabilistic flux, which measures the extent of the detailed balance broken in non-equilibrium biologically neural system. The barrier heights inferred from the landscape topography are correlated with the escape time, indicating the robustness of the decision attractor against the fluctuations. The results of barrier height also agree well with the reaction time recorded from behavioral experiments. These analyses on the landscape and transition dynamics facilitate our understanding of the underlying physical mechanism of decision making functions.

## 2. Results

**Figure 1:**
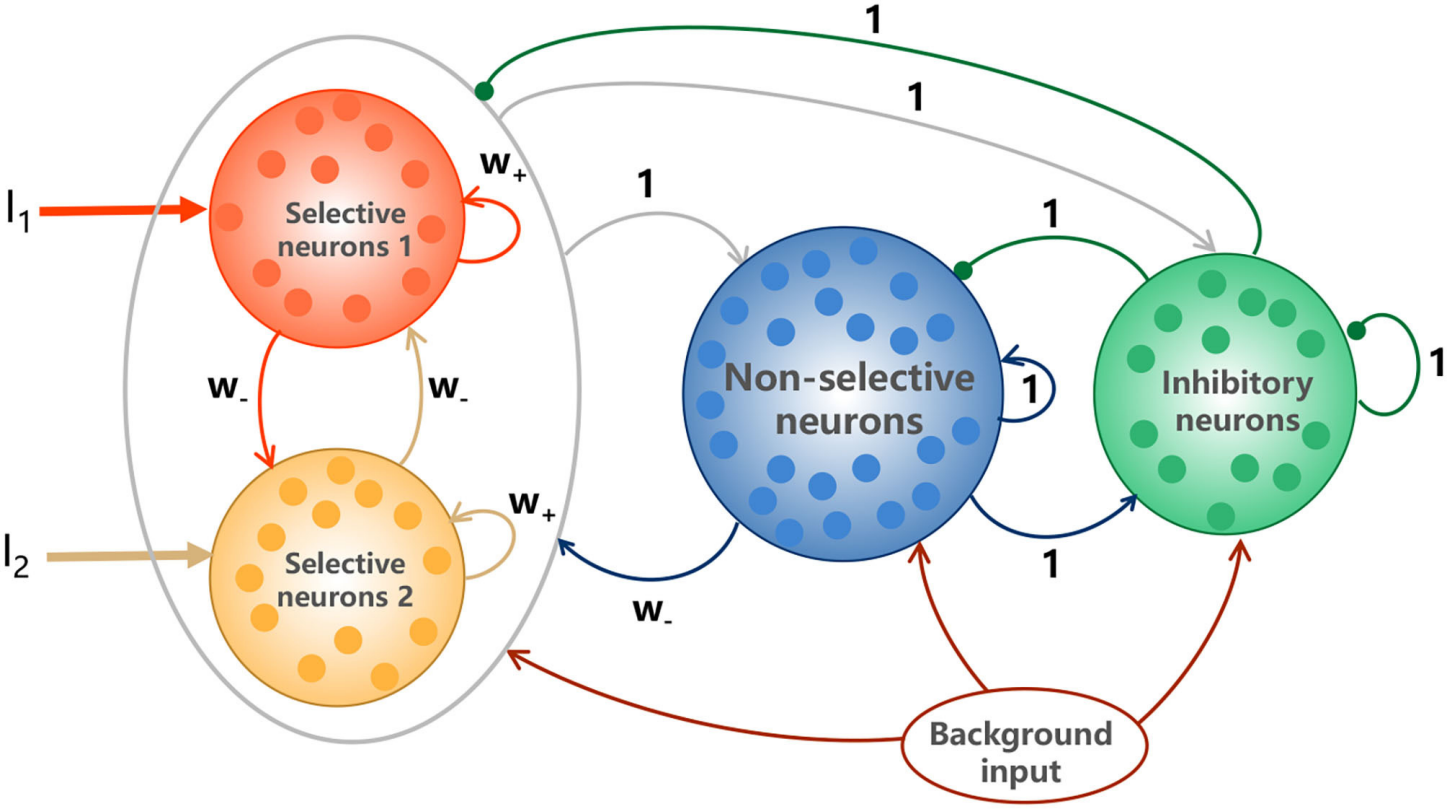
Schematic depictions of the model. The model is characterized by the strong recurrent excitation in stimulus-selective populations and the reciprocal inhibition between them mediated by GABAergic interneurons (inhibitory neurons). The two selective populations receive Poisson spike trains *I*_1_ and *I*_2_, representing the external sensory inputs. All neurons receive background inputs, allowing spontaneously firing at a few hertz. Arrows represent activation, and dots denote repression. The numbers above the links indicate the dimensionless interaction strengths.

The classic random-dot motion discrimination (RDM) task is a suitable experimental paradigm to study the perceptual decision making behavior and the associated brain activity (Newsome et al., 1989; Shadlen and Newsome, 1996, 2001; Resulaj et al., 2009; Stine et al., 2020). The monkeys are trained to judge the direction of motion in a random dot display and their choices are indicated by a saccadic eye movement. We explored a biologically realistic attractor model with spiking neurons, first introduced in Wang (2002), to account for the decision making function. The model is composed of two selective excitatory populations (labeled as S1 and S2) with each encoding one of the two target directions, one non-selective excitatory population (labeled as NS) and one inhibitory interneuron populations (labeled as I), illustrated in Figure 1. S1 and S2 are characterized by the strong recurrent self-excitations dominated by NMDA-mediated receptors and mutual inhibitions mediated by NS. The model details are illustrated in the Methods.

To imitate the sensory input of the RDM task, S1 and S2 independently receive the stimulus inputs *I*_1_ and *I*_2_ until the end of the simulation, which are modeled as uncorrelated Poisson spike trains with rates μ_1_ and μ_2_ (the unit is *Hz*). For S1, μ_1_ = μ(1+*c*), and for S2, μ_2_ = μ(1−*c*), where *c* represents the motion strength (the percentage of coherently moving dots), reflecting the difficulty of the task and μ is the stimulus strength.

In this work, we will probe how the circuit structure and stimulus input, more specifically, the recurrent connectivity *w*+ in S1 and S2, the stimulus strength μ and the motion strength *c*, influence the dynamical behavior of the decision making system in terms of the underlying energy landscape and the kinetics of state switching. The simulations are implemented on a free, open source simulator for spiking neural networks, Brain2 (Stimberg et al., 2019). And the equations (see Methods) are integrated numerically using a second order Runge-Kutta method with a time step *dt* = 0.02*ms*.

### 2.1. Multistable Landscape Quantifies the Decision Making Function

**Figure 2:**
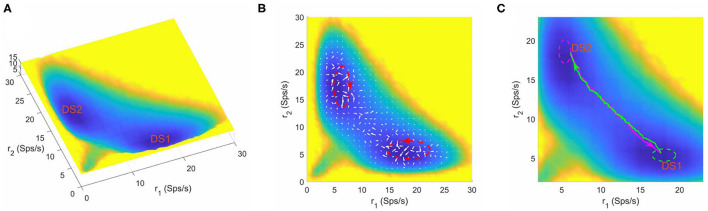
**(A)** The energy landscape for the network dynamics with bistability. **(B)** The probabilistic flux (white arrows) for the bistable system. The red dashed circles shows the curl direction of flux. **(C)** The dynamical transition paths between the two decision states for bistable dynamics. The green line is the transition path from DS1 to DS2 and the megenta line for the reverse transition. The green and meganta dashed circles indicate the decision boundaries where the corresponding decision is made. The parameters are designed as *w*+ = 1.61, μ = 58, and *c* = 0. *r*_1_ and *r*_2_ are the population averaged firing rate for the two selective pools. DS1, decision state 1; DS2, decision state 2; Sps/s, spikes/s.

In the context of a two-choice decision task, at zero coherence, the stimuli to the two selective pools are similar and hard to distinguish, so the system will make decisions randomly in a “coin tossing” manner. Therefore, it can be anticipated that there exists two symmetric attractors in favor of one of the two distinct choices (the left or right motion direction) in the binary decision system. When the parameters are specified as *w*+ = 1.61, μ = 58 and *c* = 0, we obtained a bistable attractor landscape (shown in Figure 2). The normalized steady state probability distribution *P*_*ss*_(*r*_1_, *r*_2_) is firstly quantified by collecting the statistics of the system state in the “decision space” constructed by the selective populations averaged firing rate *r*_1_ and *r*_2_, and then the potential landscape can be mapped out by *U*(*r*_1_, *r*_2_) = −*lnP*_*ss*_(*r*_1_, *r*_2_) (Sasai and Wolynes, 2003; Li and Wang, 2013a, 2014a) where *U*(*r*_1_, *r*_2_) is the dimensionless potential (see section 2 for details). Here, the potential *U*(*r*_1_, *r*_2_) does not correspond to the real energy in physical system, but a reflection of steady state probability distribution. In Figure 2A, the blue regions represent higher probability or lower potential, while the yellow regions indicate lower probability or higher potential. The landscape displays two symmetric basins of attraction. The two attractors with higher activity for one neural population (winner) and lower activity for the other (loser) are identified as distinct decision states, DS1 and DS2.

The decision making process corresponds to the transition from a spontaneous state where both S1 and S2 fire with similar low rates, to a decision state (DS1 or DS2) where they compete with each other in a winner-take-all manner, in response to the motion input. The attractor landscape provides an intuitive explanation for the decision process. Starting from an initial resting state, the system will evolve dynamically to arrive at a decision state. This process can be pictured as the system follows a path downhill to the bottom of the nearest valley which has the minimal potential energy (DS1 or DS2).

In response to the visual stimulus, subjects will make an initial decision. However, they continue to accumulate noisy evidences (Resulaj et al., 2009) or monitor the correctness of the previous decision (Cavanagh and Frank, 2014) after a choice has been made. Then the decision-maker may or may not change the mind, i.e., the initial decision is made to subsequently either reverse or reaffirm. Since the decision-making is endowed with randomness from the external stimulus and spontaneous activity fluctuations, the stability of an attractor state in landscape is only guaranteed up to a limited time-scale. In the case of changing minds, the noise will terminate the self-sustained pattern of activity and drive the system to escape away from the initial attractor and switch to the other although the stimulus input remains unchanged.

The neural system operates far from equilibrium. So we quantified the non-equilibrium probability flux map (denoted by the white arrows in Figure 2B) to measure the extent of the violation of detailed balance. The flux is calculated from the dynamical stochastic trajectories of neural activity (the time window used to calculated firing rate is 50*ms*) over a long time (see section 2) (Battle et al., 2016). We can see that the force from the curl flux drives the system away from one local attractor and guides the transition to the other. To see the effects of using different time windows for simulations, we showed typical trajectories for using different time windows (Supplementary Figure 1). We also estimated the landscape and probabilistic flux when the time window used to calculate firing rate is 20*ms* (Supplementary Figure 2). A major difference for different time windows is that for smaller time windows there are larger fluctuations (Supplementary Figure 1), which is also reflected by the landscape results showing that the landscape using 20*ms* as time window displays a little more variations (compare Figure 2B and Supplementary Figure 2). As for the curl flux, different time windows lead to qualitatively similar results (compare Figure 2B and Supplementary Figure 2), i.e., the magnitude of flux is larger in the region close to the basins, and the flux has certain curl direction.

The dynamical transition paths corresponding to the changes of mind process (green line for DS1 to DS2 and megenta line for DS2 to DS1) are shown in Figure 2C. Note the fact that the forward and backward paths are irreversible, which is the consequence of the non-equilibrium curl flux (Wang et al., 2008; Li and Wang, 2014a; Yan and Wang, 2020). It is worth noting that the curl flux and noise play different roles in the decision making system. Here, the role of the none-zero flux is breaking detailed balance, which is also the cause for the irreversibility of forward and backward transition paths (the green path and the magenta path are not the same, Figure 2C). If the flux is zero, the green and magenta path will duplicate completely, which corresponds to an equilibrium case (Wang, 2015). To see whether this irreversibility is due to stochastic effects, we used different stochastic trajectories to calculate transition path (Supplementary Figure 3) and obtained consistent curl directions for the irreversible transition paths for both bistable and tristable system. We also showed single-trajectory examples, which support the irreversibility for the transition path for both bistable and tristable cases (Supplementary Figure 4). Therefore, flux is important for the state transitions. In terms of the role of noise, we see that although the landscape and flux keep the same, the single sample for transition path varies due the noise effects (Supplementary Figure 4). The noise also contributes to the state transition for the barrier crossing process.

**Figure 3:**
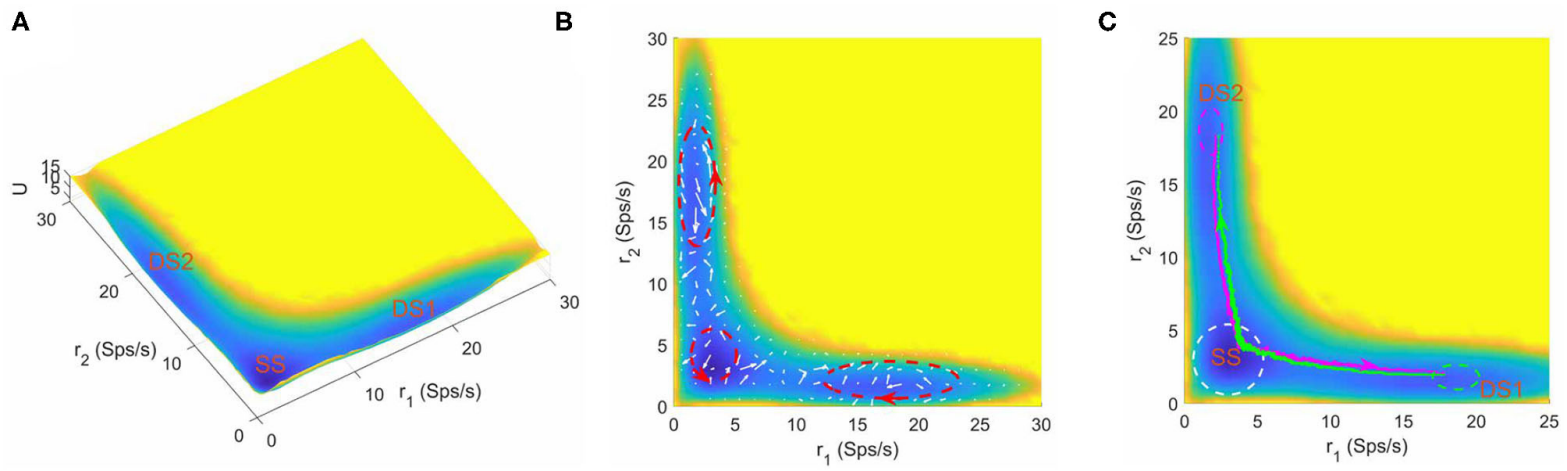
**(A)** The energy landscape for network dynamics with tristability. **(B)** The probabilistic flux (white arrows) for tristable system. The red dashed circles show the curl direction of flux. **(C)** The dynamical transition paths between the two decision states. The green line is the transition path from DS1 to DS2 and the megenta line for the reverse transition. The change of mind process evolves through the SS state. The green and meganta dashed circles indicate the decision boundaries where the corresponding decision is made and the white dashed circle represents the attractor region for spontaneous state. The parameters are calibrated as *w*+ = 1.66, μ = 16, and *c* = 0. *r*_1_ and *r*_2_ are the population averaged firing rate for the two selective pools. SS, spontaneous state, DS1, decision state 1; DS2, decision state 2; Sps/s, spikes/s.

Furthermore, we found that the number of stable attractors on landscape changes under different parameter combinations. Figure 3A shows the tristable attractor landscape of the decision making network for *w*+ = 1.66, μ = 16 and *c* = 0. Compared to Figure 2A, the attractor that emerges at the bottom left corner is identified as the spontaneous undecided state with low activities of both selective neural populations, indicated by SS on the landscape. Similar to the case of bistability, the property of curl flux and irreversibility of transition path also exist in the tristable system (Figures 3B,C), illustrating the inherent non-equilibrium property of neural system. It is worth noting that the kinetic switches between two decision states go through the SS state (green and megenta path in Figure 3C), indicating that the system will erase the former decision (back to the resting state) firstly and then make another decision (Pereira and Wang, 2014). So the SS state can be treated as an intermediate state.

The intermediate state has been observed in various biological process. For example, in epithelial–mesenchymal transition, stem cell differentiation and cancer development, the intermediate state cell types play crucial roles in cell fate decision system governed by corresponding gene regulatory networks (Lu et al., 2013; Li and Wang, 2014b; Li and Balazsi, 2018; Kang et al., 2019). For working memory, the presence of the intermediate state significantly enhances the flexibility of the system to a new stimulus without seriously reducing the robustness against distractors (Yan and Wang, 2020). For the decision making function in this work, our landscape picture provides some hints on the roles of the intermediate state on mind changing. The mind changing process occurs in a step-wise way with the existence of the SS state. The system will switch to SS state firstly and stay there for a while, and then depending on the new accumulated evidences, decide whether transit to the other decision state (change of minds) or return back to the initial decision state (reaffirm previous decision) (Figure 3C). This demonstrates that the intermediate state may increase plasticity and robustness of perceptual decision making as the system can switch back to the original decision state from intermediate state if the accumulated evidences are not enough for change of mind.

### 2.2. Stimulus Strength Influences Decision Making Process by Altering Landscape Topography

**Figure 4:**
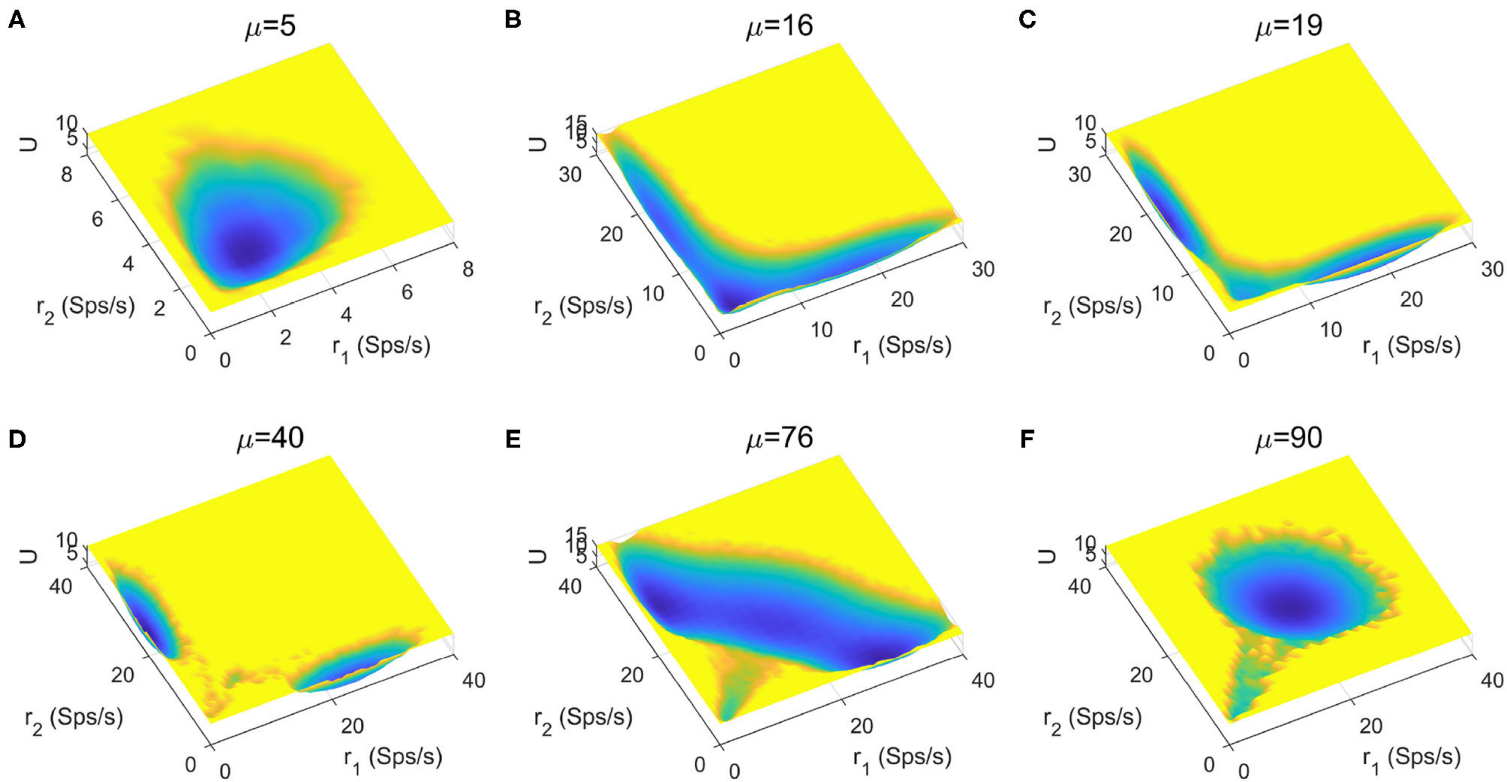
The energy landscape over a range of external inputs, applied symmetrically to both selective pools (0% coherence). **(A)** With slight stimulus, none of the decided attractors can be reached, the network stays at the resting state. **(B)** Two attractors corresponding to the decision states emerge for larger stimulus strength. **(C)** The central basin of the spontaneous state becomes weaker for increasing stimulus strength. **(D)** When the stimulus input is strong enough, the resting state vanishes, and the two decision states remain on two sides. **(E)** The strong inputs induce the emergence of an intermediate state, called “double-up” state, with high activity for both selective neural assemblies between decision states. **(F)** The “double-up” state becomes the exclusively steady state while two symmetric decision states disappear simultaneously for the further increase of the stimulus strength.

The stimuli strength applied to the two selective pools is a key factor in decision making tasks. In Figure 4, we quantitatively mapped out the potential landscape for the decision-making dynamics under different stimulus strength μ. The landscape displays three qualitatively different topographies (monostable, bistable and tristable) across the range of stimuli strength from 5 to 90*Hz*.

For small inputs, the spontaneous state with both selective populations firing at low rates, is the exclusively stable state (Figure 4A). The additional increase of stimulus strength induces the emergence of the two decision attractors (Figure 4B). The central basin of the spontaneous state becomes weaker for increasing stimulus strength (Figure 4C) until the system operates in a binary decision-making region (Figure 4D). When the selective inputs are sufficiently high, an intermediate state with high activity for both neural assemblies encoding the possible alternatives (Figure 4E), called "double-up" state, emerges, in line with both experimental evidences and computational results in delay response tasks (Shadlen and Newsome, 1996; Roitman and Shadlen, 2002; Huk and Shadlen, 2005; Wong and Wang, 2006; Martí et al., 2008; Albantakis and Deco, 2011; Yan et al., 2016; Yan and Wang, 2020). The “double-up” state reduces the barrier between decision states and the transitions are more likely to occur, facilitating changes of mind (Albantakis and Deco, 2011; Yan and Wang, 2020). The two symmetric decision states disappears simultaneously for the further increase of the stimulus strength, so the system loses its ability to compute a categorical choice (Figure 4F).

**Figure 5:**
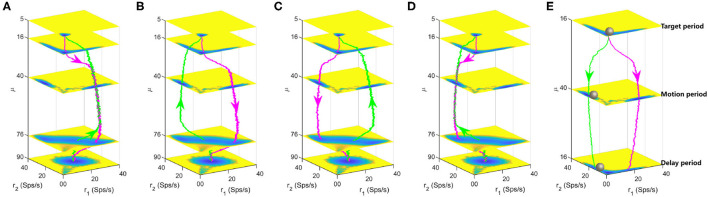
Landscapes are shown in the four-dimensional pictures. The magenta and green lines are the dynamical decision paths averaged over many trials with continuously varied stimulus input along the *z* axis. Each layer corresponds to a three-dimensional landscape with fixed stimulus strength μ. **(A)** The paths for increasing and decreasing input both pass through the DS1 state. **(B)** The paths for increasing and decreasing input pass through the DS1 and DS2 states, respectively. **(C)** The paths for increasing and decreasing input pass through the DS2 and DS1 states, respectively. **(D)** The paths for increasing and decreasing input both pass through the DS2 state. **(E)** A quantitative landscape to illustrate the maintenance of decisions during the delay period in the delayed version of random-dot motion discrimination task. The gray balls represent the instantaneous states of the system. The parameters are specified as *w*+ = 1.66 and *c* = 0.

To track the landscape change and dynamical transition property of decision-making process, we calculated the typical kinetic transition paths averaged over many trials for continuously increasing (from 5 to 90*Hz*, magenta lines) and decreasing (from 90 to 5*Hz*, green lines) stimulus inputs, as displayed in Figures 5A–D. There exists four combinations and corresponding landscapes of the decision path since the forward and backward paths can pass through either the DS1 or the DS2 state due to the stochasticity. In Figures 5A,D, the backward path deviates away from the forward decision-making path due to the non-equilibrium flux force although the two paths pass through the same decision state (DS1 or DS2). Figures 5B,C show the forward and backward paths pass through distinct decision states. It can be seen that the system will spend some time staying at the spontaneous state (μ = 5, 16) before making a decision (μ = 40) for the forward paths while maintaining the "double-up" state for a period of time (μ = 90, 76) before making a decision (μ = 40) for the backward paths, implying the existence of hysteresis for state switches in biological neural system.

The energy landscape we obtained here also explains the delayed version of the RDM task well, which involves both the decision computation and working memory. The delayed RDM task additionally requires the subjects to withhold the choice in working memory across a delay period before responding to the saccadic eye movement (Shadlen and Newsome, 2001). Figure 5E shows the three layers of landscape corresponding to the targets, motion and delay in the delayed RDM task, respectively. The gray balls represent the instantaneous states of the system. The megenta and green lines are two possible decision paths due to the symmetry and fluctuations. Initially, the system rests at the spontaneous state until the visual inputs force the system switching into one of the two decision attractor states. The decision is stored in the DS1 or DS2 and this information will be retrieved to produce motor responses at the end of the delay period even though the stimulus is absent. This quantitative picture of landscape echos with the one-dimensional illustrative diagram of decision “landscape” proposed in Wong and Wang (2006).

**Figure 6:**
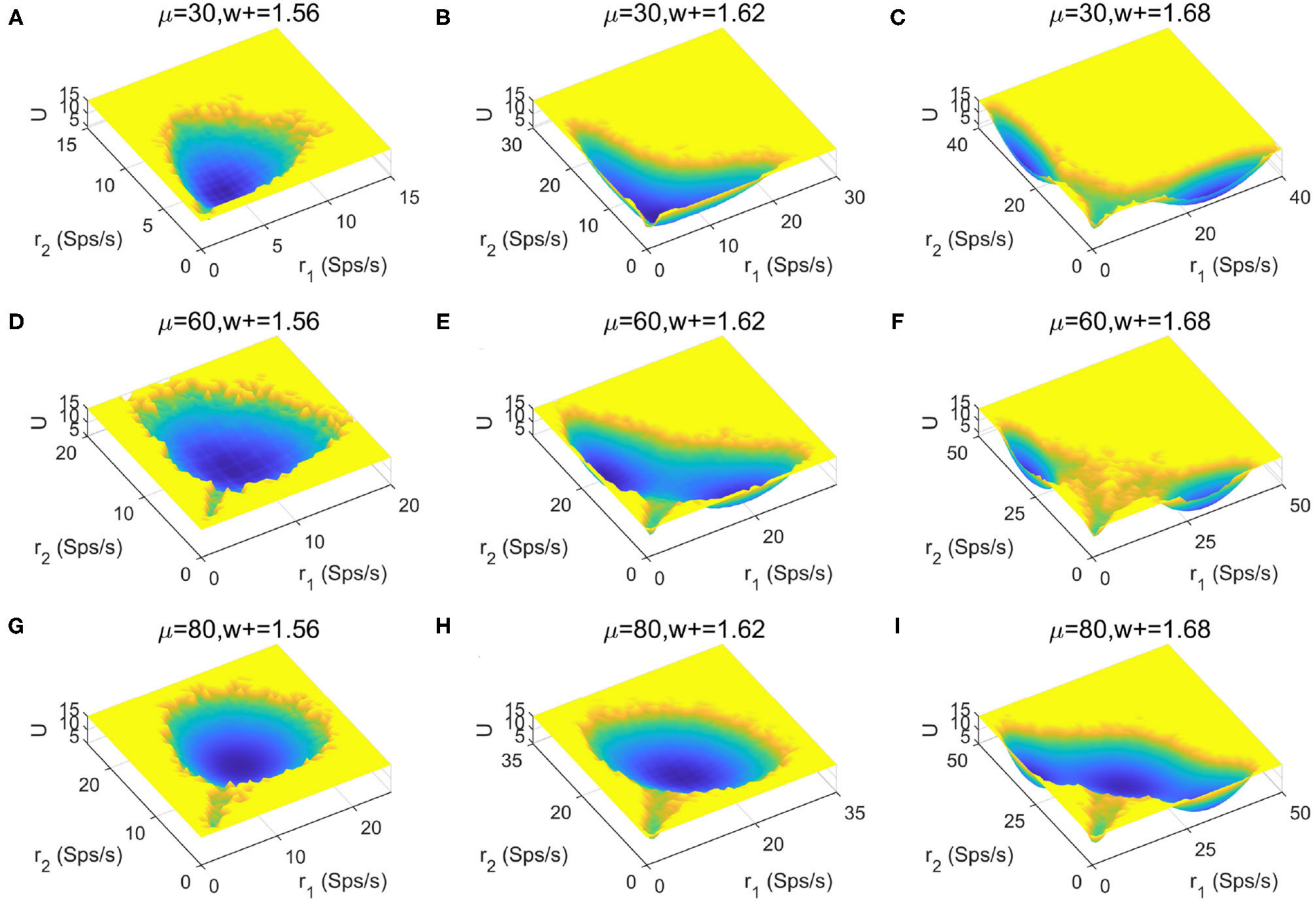
Landscape comparisons when the stimulus strength μ and recurrent connectivity in the two selective populations *w*+ change.

To provide a more global picture of the system dynamics, we explored the change of landscape topography when the stimulus strength μ and recurrent connectivity in the two selective populations *w*+ vary (Figures 6A–I). We found that the number of stable states increases as the recurrent connectivity *w*+ increases (horizontal direction), while the stimulus strength μ increases (vertical direction) there is no apparent trend for the occurrence of multistability. This suggests that stronger self-activation strength may promote the occurrence of multistability.

### 2.3. The Influence of the Difficulty of Task on Landscape Stability

The stability of attractor states is critical for decision-making network since the decisions can be changed easily for unstable decision state. Experimental and modeling works both suggested that the probability of changes of mind depends on the task difficulty, namely, the motion strength *c* (Resulaj et al., 2009; Albantakis and Deco, 2011). For biased stimulus input (*c*≠0), the symmetry of attractor landscape is broken (Supplementary Figures 5, 6 for the asymmetric landscapes). Correspondingly, we can calculate relative barrier height (RB) between pairs of local minima and mean first passage time (MFPT) to quantitatively measure the global stability of the neural network under different motion strength *c*.

For bistable system, the relative barrier height (*RB*_1_) is defined in terms of the two basins of decision state. Based on landscape topography, we define *U*_1_ and *U*_2_ as the potential minimum of the DS1 and DS2, and *U*_*saddle*_ as the potential of the saddle point between the two states. The barrier heights quantifying the global stability are *BH*_1_ = *U*_*saddle*_ − *U*_1_ and *BH*_2_ = *U*_*saddle*_ − *U*_2_. Then *RB*_1_ = *BH*_1_ − *BH*_2_, quantifying the relative stability of DS1 against DS2.

The MFPT represents the average kinetic time of the alternations between different attractor states, thus also describing the stability of attractors. With dynamical neural activity trajectories over a long time window, we can estimate the time taken for the system to switch from one attractor to another for the first time and get the first passage time (FPT). Here, an attractor region is roughly taken as a small ellipse centered on the local minimum (megenta and green dashed circles in Figure 2C), and the transition is finished once the stochastic trajectory enters the destination ellipse. Then the MFPT is defined as the average of FPT by samplings.

**Figure 7:**
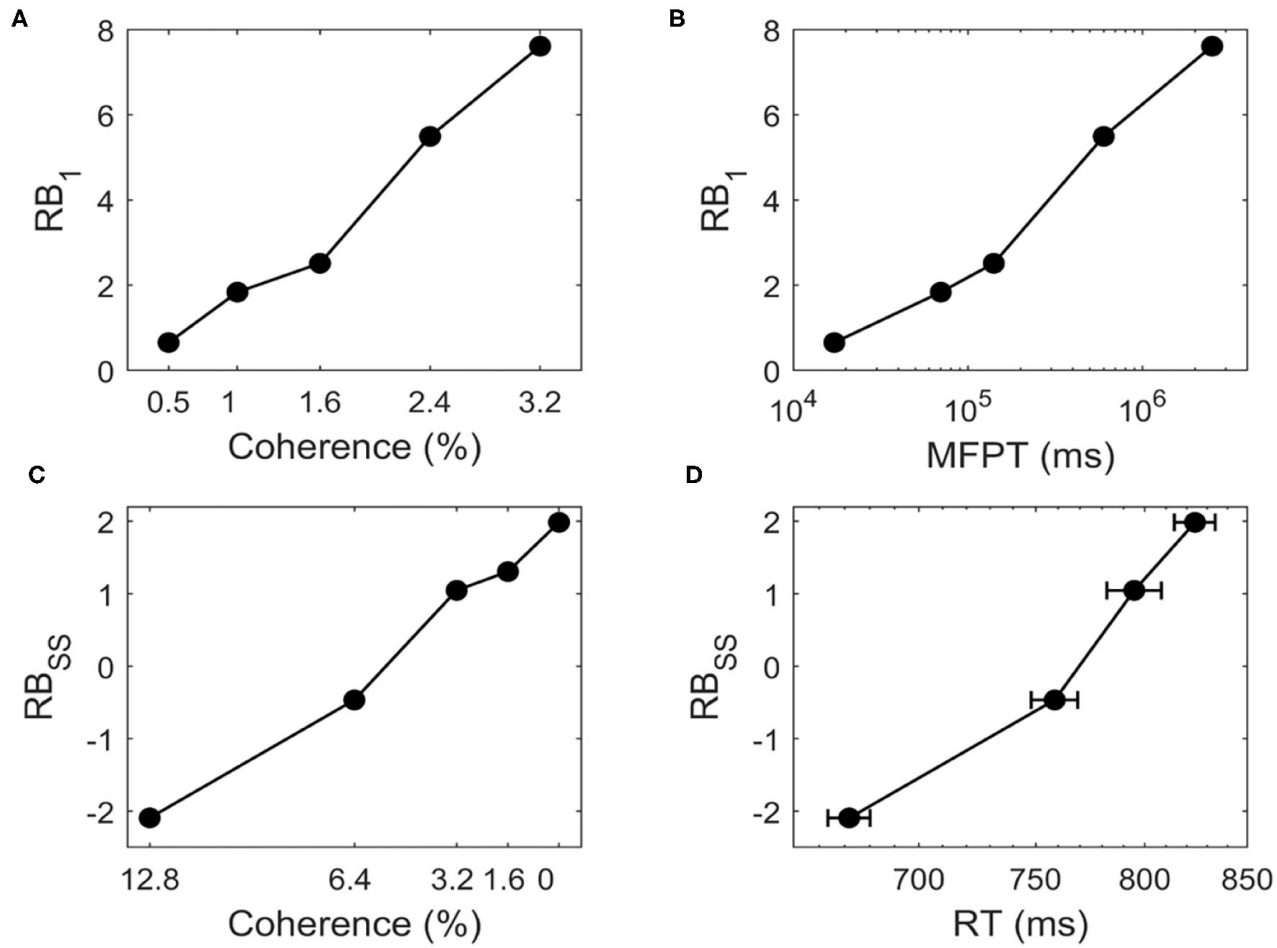
For the change of mind process from bistable landscape, **(A)** the relative barrier height (*RB*_1_) increases for *c* = 0.5%, 1%, 1.6%, 2.4%, 3.2%, **(B)** *RB*_1_ is correlated with the logarithm of the mean first passage time (MFPT). *RB*_1_ represents relative barrier height from DS1 to DS2, which quantifies the global stability of DS1 against DS2. The MFPT from DS1 to DS2 are estimated from dynamical neural activity trajectories. For the decision making process from tristable landscape, **(C)** The relative barrier height (*RB*_*SS*_) increases for *c* = 12.8%, 6.4%, 3.2%, 0%, **(D)** *RB*_*SS*_ is correlated with the logarithm of the reaction time (RT, mean ± SEM). *RB*_*SS*_ denotes the relative barrier height for the transition from SS to DS1. *RT* is the experimental observed reaction time of correct trials in the motion-discrimination task (Roitman and Shadlen, 2002).

We study whether the landscape can quantify the transition time. Figure 7A shows that for bistable system, *RB*_1_ increases as we increase *c* for *c* = 0.5%, 1%, 1.6%, 2.4%, 3.2%. The positive value of *RB*_1_ indicates that the two stable states are no longer symmetric and the DS1 has a deeper basin of attraction than DS2 due to the biased external inputs. When the motion strength gets stronger (larger *c*), the time taken to switch from DS1 to DS2 increases with higher barrier (Figure 7B), indicating that when the task is easier (larger *c*) the change of mind needs more time, i.e., the change of mind (the right decision becomes wrong decision in current case) is harder.

We further ask how the model predictions from landscape fit quantitative experimental data. We use tristable system as an example to describe the decision process. Here, the relative barrier height (*RB*_*SS*_) between SS and DS1 has the similar definition as *RB*_1_. Figure 7C shows that for tristable system, *RB*_*SS*_ increases as we decrease *c* for *c* = 12.8%, 6.4%, 3.2%, 0%, indicating that it is harder to make a decision since the barrier is higher for the more difficult task. The transition from SS to DS1 represents the decision process, thus related to the behavioral reaction time (RT). The positive correlation between *RB*_*SS*_ and the experimentally observed *RT* (Roitman and Shadlen, 2002) suggests that the speed of decisions decreases in harder decision tasks (smaller *c*) with higher barrier (Figure 7D). Intuitively, this means that for a more difficult task, it is harder to make decision, which takes longer reaction time. Therefore, the landscape results explain the experimental data well and provide an intuitive and quantitative way to understand the decision making function.

Of note, the linear correlation between the logarithm of MFPT and RT and the barrier height of landscape is not a perfect fit. In fact, this is due to another important factor, the curl flux. For a non-equilibrium system, the landscape may explain a major part of the transition rate (barrier crossing from a potential valley). However, the landscape does not solely determine the transition rate as in an equilibrium system, i.e., the flux will contribute to the transition dynamics for barrier crossing (Feng et al., 2014). We should notice that due to the approximation nature of this approach, at very small or very large time scales, it might be less precise for the estimation of the relationship between MFPT and barrier height.

## 3. Discussion

The biological neural circuit underlying the decision-making function is a non-linear, non-equilibrium, non-stationary, open and strongly coupled system. The underlying energy landscape allows us to understand the complex dynamic behavior of the neural system from a global view. In this work, we explicitly quantified the landscape and further studied the stochastic transition dynamics for perceptual decision making from the plausible biophysical spiking cell-based model to mimic the visual motion discrimination task.

When the motion strength *c* is zero, i.e., the two selective populations receive stimuli with the same strength, we identified qualitatively different landscape topographies with different number of attractors in the phase space under certain parameter regions. The bistable attractor landscape is characterize by two symmetric basins of attraction corresponding to the two competing decision states. The spontaneous state with both selective pools firing at low rates, as an intermediate state, emerges on tristable attractor landscape, which increases the plasticity by making a two-step transition for change of mind and robustness by reaffirming previous decision of decision making. The irreversibility of the bistable and tristable switches due to the probabilistic curl flux demonstrates the inherent non-equilibrium property of the neural decision system. We found that the neural ensembles evolve across different regimes under the control of driving input. Of note, a new intermediate state, the "double-up" state with both selective pools firing at high rates also emerges when the stimulus strength is sufficiently high. The "double-up" state reduces the barrier between the two decision states and facilitates the state transitions, in line with both experimental evidences and computational results in delay response tasks (Shadlen and Newsome, 1996; Roitman and Shadlen, 2002; Huk and Shadlen, 2005; Wong and Wang, 2006; Albantakis and Deco, 2011; Yan et al., 2016; Yan and Wang, 2020). By exploring the parameter region, we found that a possible way of promoting the occurrence of mutilstability and intermediate state is to increase the strength of recurrent connectivity or self-activation of neural populations.

We also quantified the global stability of decision-making by barrier height and mean first passage time to explore the influence of the difficulty of task. When the difficulty of task increases, namely, the coherence level *c* decreases, the speed of decisions gets slower and it takes longer for the system to make a decision characterized by higher barrier height, which is consistent with experimental observations. The landscape and path results advance our understanding of stochastic dynamical mechanism of decision-making function.

In previous study, Wong and Wang reduced the spiking neural network model to a two-variable coupled ordinary differential equations by mean-field approximations (Wong and Wang, 2006). This allows the analysis of dynamics with the tools of nonlinear dynamical system, such as phase-plane analysis and bifurcation based on ordinary differential equations. However, these analysis is under the framework of deterministic simulation, so the stochastic dynamics is hard to be studied from bifurcation type of approaches. The non-equilibrium potential theory we employed here is characterized by its ability to quantify the stochastic dynamics and global stability of decision-making function. The barrier height inferred from potential landscape and mean passage time measure the difficulty of switching between basins of attractions, which is also applicable in large-scale model. The quantitative picture of landscape in Figure 5E also echos with the one-dimensional illustrative diagram of decision “landscape” proposed in Wong and Wang (2006).

The spiking neural network we studied here consists of thousands of neurons interacting in a highly nonlinear manner, which is more biologically realistic compared to simplified firing rate-based model. Each parameter in the model has specific biologically meaning, allowing us to explore the neural mechanisms of decision-making. However, there are also some limitations for current spiking neural network model. Firstly, the numerical simulation of spiking neural network is computationally intensive and time-consuming. The accuracy of our results depends on the volume of collected dynamical trajectories, which is limited by the computational efficiency. Secondly, to facilitate our analysis, we reduced the dimension of spiking neural network system to 4 by averaging the microscopic activity of individual neurons in homogeneous populations to obtain macroscopic population-averaged firing rate. For visualization and analysis, we projected the system to the two dimensions (*r*_1_ and *r*_2_) for the estimation of potential landscape and flux. This may introduce inaccuracy for analyzing original high-dimensional system, quantitatively. So, it's also important to develop other dimensional reduction approach (Kang and Li, 2021). Furthermore, the local circuit model we studied here only involves individual brain area, while a biologically realistic decision-making may be distributed, engaging multiple brain regions (Siegel et al., 2015; Steinmetz et al., 2019). It is anticipated that the landscape and path approach can be applied to account for other cognitive function related issues (such as distributed working memory or decision-making) in more realistic brain networks considering more complex circuits in neural network scale or brain region scale (Murray et al., 2017; Schmidt et al., 2018; Mejias and Wang, 2021).

## 4. Methods

### 4.1. Neural Network Model

The biologically plausible model for binary decision making was first introduced in Wang (2002), illustrated in Figure 1. The model is a fully connected network, composed of *N* neurons with *N*_*E*_ = 1600 for excitatory pyramidal cells and *N*_*I*_ = 400 for inhibitory interneurons, which is consistent with the observed proportions of the pyramidal neurons and interneurons in the cerebral cortex (Abeles, 1991). Two distinct populations in excitatory neurons (S1 and S2) respond to the two visual stimuli, respectively, with *N*_*S*1_ = *N*_*S*2_ = *fN*_*E*_ and *f* = 0.15, and the remaining (1−2*f*)*N*_*E*_ non-selective neurons (NS) do not respond to either of the stimuli. All neurons receive a background Poisson input of *v*_*ext*_ = 2.4*kHz*. This can be viewed as originating from 800 excitatory connections from external neurons firing at 3*Hz* per neuron, which is consistent with the resting activity observed in the cerebral cortex (Rolls et al., 1998).

Table 1 is the connection weight matrix between these four populations, where minus sign represents inhibition effect. The synaptic weights between neurons are prescribed according to the Hebbian rule and remain fixed during the simulation. Therefore, inside the selective populations, the synapses are potentiated and the weight *w*+ is larger than 1. Between selective populations and from nonselective population to selective ones, the synaptic weight is *w*_−_ = 1 − *f*(*w*_+_ − 1)/(1 − *f*) so that the overall recurrent excitatory synaptic drive in the spontaneous state remains constant when altering *w*+ (Amit and Brunel, 1997). *w*_−_ < 1 indicates the synaptic repression. The remaining weights are 1.

**Table 1:**
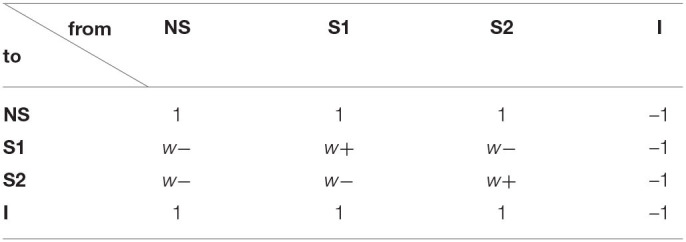
Connection weight matrix.

Herein, we consider the leakage integrate-and-fire (LIF) model to describe both pyramidal cells and interneurons (Tuckwell, 1988). The membrane potential of a neuron, *V*(*t*), can be described by a capacitance-voltage (CV) equation when it is less than a given voltage threshold *V*_*th*_,


(1) $$\begin{array}{l} C_{m}\frac{dV\left(t\right)}{dt}=-g_{L}\left(V\left(t\right)-V_{L}\right)-I_{syn}\left(t\right),\;V\leq V_{th}. \end{array}$$

Here, *C*_*m*_ is the capacitance of the neuron membrane, *C*_*m*_ = 0.5*nF* (0.2*nF*) for excitatory (inhibitory) neurons. *g*_*L*_ is the leakage conductance, *g*_*L*_ = 25*nS* (20*nS*) for excitatory (inhibitory) neurons. Each neuron has a leakage voltage *V*_*L*_ = −70*mV* and firing threshold *V*_*th*_ = −50*mV*. *I*_*syn*_ represents the total synaptic current flowing into the neuron.

When *V*(*t*) = *V*_*th*_ at *t* = *t*_*k*_, the neuron will emit a spike and the membrane potential is reset at *V*_*reset*_ = −55*mV* for a refractory period τ_*ref*_,


(2) $$\begin{array}{l} V\left(t\right)=V_{reset},\;t\in \left[t_{k},t_{k}+\tau_{ref}\right]. \end{array}$$

After then, *V*(*t*) is governed by the CV (Equation 1) again. Here, τ_*ref*_ = 2*ms* (1*ms*) for excitatory (inhibitory) neurons.

The synaptic model maps the spike trains of the presynaptic neuron to the postsynaptic current. For the fully connected neural network, the total postsynaptic current is the sum of the following four currents:


(3) $$\begin{array}{l} I_{syn}\left(t\right)=I_{ext,AMPA}\left(t\right)+I_{rec,AMPA}\left(t\right)+I_{rec,NMDA}\left(t\right)+I_{rec,GABA}\left(t\right) \end{array}$$

The first term is the external excitatory current, which is assumed to be exclusively mediated by AMPA receptor. The second and third terms are the recurrent excitatory currents mediated by AMPA and NMDA receptors. The last term is the inhibitory current mediated by GABA receptor. More specifically,


(4) $$\begin{array}{l} \begin{array}{c} I_{ext,AMPA}\left(t\right)=g_{ext,AMPA}\left(V\left(t\right)-V_{E}\right)s^{\text{ext},\text{AMPA}}\left(t\right)\\ I_{rec,AMPA}\left(t\right)=g_{rec,AMPA}\left(V\left(t\right)-V_{E}\right)\overset{N_{E}}{\underset{\text{j}=1}{\sum }}w_{j}s_{j}^{\text{AMPA}}\left(t\right)\\ I_{rec,NMDA}\left(t\right)=\frac{g_{NMDA}\left(V\left(t\right)-V_{E}\right)}{\left(1+\left[Mg^{2+}\right]\exp\left(-0.062V\left(t\right)\right)/3.57\right)}\overset{N_{E}}{\underset{j=1}{\sum }}w_{j}s_{j}^{NMDA}\left(t\right)\\ I_{rec,GABA}\left(t\right)=g_{GABA}\left(V\left(t\right)-V_{I}\right)\overset{N_{I}}{\underset{j=1}{\sum }}s_{j}^{GABA}\left(t\right), \end{array} \end{array}$$

where *V*_*E*_ = 0*mV*, *V*_*I*_ = −70*mV*. For excitatory cells, the synaptic conductances for different channels are *g*_*ext, AMPA*_ = 2.1*nS*, *g*_*rec, AMPA*_ = 0.05*nS*, *g*_*NMDA*_ = 0.165*nS*, and *g*_*GABA*_ = 1.3*nS*; for inhibitory cells, *g*_*ext, AMPA*_ = 1.62*nS*, *g*_*rec, AMPA*_ = 0.04*nS*, *g*_*NMDA*_ = 0.13*nS*, and *g*_*GABA*_ = 1.0*nS*. *w*_*j*_ is the dimensionless synaptic weight and *s*_*j*_ is the gating variable, representing the fraction of open channels for different receptors. The sum over *j* denotes a sum over the synapses formed by presynaptic neurons *j*. Specially, the NMDA synaptic currents depend on both the membrane potential and the extracellular magnesium concentration ([Mg^2+^] = 1*mM*) (Jahr and Stevens, 1990). The gating variables are given by


(5) $$\begin{array}{l} \begin{array}{c} \frac{ds_{j}^{AMPA}\left(t\right)}{dt}=-\frac{s_{j}^{AMPA}\left(t\right)}{\tau_{AMPA}}+\underset{k}{\sum }\delta \left(t-t_{j}^{k}\right)\\ \frac{ds_{j}^{NMDA}\left(t\right)}{dt}=-\frac{s_{j}^{NMDA}\left(t\right)}{\tau_{NMDA,decay}}+\alpha x_{j}\left(t\right)\left(1-s_{j}^{NMDA}\left(t\right)\right)\\ \frac{dx_{j}\left(t\right)}{dt}=-\frac{x_{j}\left(t\right)}{\tau_{NMDA,rise}}+\underset{k}{\sum }\delta \left(t-t_{j}^{k}\right)\\ \frac{ds_{j}^{GABA}\left(t\right)}{dt}=-\frac{s_{j}^{GABA}\left(t\right)}{\tau_{GABA}}+\underset{k}{\sum }\delta \left(t-t_{j}^{k}\right) \end{array} \end{array}$$

where the decay time for AMPA, NMDA and GABA synapses are τ_*AMPA*_ = 2*ms*, τ_*NMDA, decay*_ = 100*ms* and τ_*GABA*_ = 5*ms* (Hestrin et al., 1990; Spruston et al., 1995; Salin and Prince, 1996; Xiang et al., 1998). The rise time for NMDA synapses are τ_*NMDA, rise*_ = 2*ms* (the rise times for AMPA and GABA are neglected because they are typically very short) and α = 0.5*ms*^−1^ (Hestrin et al., 1990; Spruston et al., 1995). The sum over *k* represents the sum over all the spikes emitted by presynaptic neuron *j* at time $t_{j}^{k}$. For external AMPA currents, the spikes are generated by independently sampling Poisson process with rate *v*_*ext*_ = 2.4*kHz* from cell to cell.

### 4.2. Potential Landscape and Probabilistic Flux Theory

By numerically simulating the neural network system for a long time, we can obtain the raster plot, i.e., neurons emit spikes at specific time points. However, since the spiking neural network we studied here is a high dimensional system consisting of thousands of interacting neurons, we focus on the macroscopic activity of population-averaged firing rate rather than microscopic neural spikes, which effectively reduced the dimension of the system dynamics to 4 (4 populations). For visualization, two more important dimensions, i.e., the population-averaged firing rates of two selective neural groups *r*1 and *r*2 are chosen as coordinates to form the "decision space" and then the probablistic distribution at steady state (energy landscape) is projected into the decision space by integrating other dimensions. *r*_1_ and *r*_2_ can be calculated by firstly counting the total spike numbers of a population in a time window of 50*ms*, which slides with a time step of 5*ms*, and then dividing it by the neuron number and the time window to get *r*_1_ and *r*_2_.

To visualize the probability distribution of the system state in the "decision space" constructed by the firing rate *r*_1_ and *r*_2_, we discrete the space into a collection of grids and collect the statistics for the system state falling into each grid. Finally, the potential landscape is mapped out by *U*(*r*_1_, *r*_2_) = −*lnP*_*ss*_(*r*_1_, *r*_2_) (Sasai and Wolynes, 2003; Li and Wang, 2013a, 2014a), where *P*_*ss*_(*r*_1_, *r*_2_) represents the normalized joint probability distribution at steady state and *U*(*r*_1_, *r*_2_) is the dimensionless potential.

Of note, a key issue is to decide when a stationary distribution has been reached. Theoretically, the steady state distribution need to be obtained as time *t* goes to infinity (or very large). Since the numerical simulation of spiking neural network is computationally intensive and time-consuming, the time length of the dynamical trajectory we can obtained is limited. To address this problem, we define relative Euclidean distance between two probability distributions as $\sigma =\sqrt{\frac{\underset{ij}{\sum }{\left(P_{ij}^{\text{t+500}}-P_{ij}^{t}\right)}^{2}}{{\left(\underset{ij}{\sum }P^{\text{t}}\right)}^{2}}}$, where $P_{ij}^{t}$ and $P_{ij}^{t+500}$ are probability distributions obtained by firing rate activity with time length *t* and *t*+500, respectively. So σ measures the deviation of distribution by prolonging the trajectory by 500*s*. If increasing time *t* does not significantly change this relative distance for the probability distribution between different time length (σ is less than a threshold, σ < 0.06% for bistable landscape in Figure 2), we consider that a steady state has been reached.

Biological systems, including neural circuits, are generally dissipative, exchanging energies or materials with the environment to perform functions (Lan et al., 2012). For a non-equilibrium system, the violation of detailed balance lies at the heart of its dynamics. Different from the equilibrium system whose dynamics is solely determined by the underlying energy landscape, the non-equilibrium system is also driven by the steady state probabilistic flux, which measures to what extent the system is out of equilibrium or the detailed balance is broken (Wang et al., 2008; Yan et al., 2013, 2016; Li and Wang, 2014a; Yan and Wang, 2020). For high-dimensional systems, for example, the neural network we studied here, it is challenging to quantify the non-equilibrium probabilistic flux from diffusion equation (Wang et al., 2008; Li and Wang, 2014a). Therefore, we employ an approach which is based on the fluctuating steady-state trajectories, to quantify the probability flux (Battle et al., 2016). As we discussed before, the reduced stochastic system trajectory evolves over time in a four-dimensional phase space. However, for the sake of simplicity and visualization, the two more important dimensions, *r*_1_ and *r*_2_, are utilized to estimate the probabilistic flux. To determine the probability flux of the non-equilibrium neural system, we discrete the subspace constructed by *r*_1_ and *r*_2_ in a coarse-grained way, i.e., the subspace is divided into *N*_1_ × *N*_2_ equally sized, rectangular boxes, each of which represents a discrete state α. Such a discrete state is a continuous set of microstates. Then the probability flux associated with state α is the following vector:


(6) $$\begin{array}{l} \vec{J}\left({\vec{x}}_{\alpha }\right)=\frac{1}{2}\left(\begin{array}{c} w_{\alpha^{-},\alpha }^{\left(r_{1}\right)}+w_{\alpha ,\alpha^{+}}^{\left(r_{1}\right)}\\ w_{\alpha^{-},\alpha }^{\left(r_{2}\right)}+w_{\alpha ,\alpha^{+}}^{\left(r_{2}\right)} \end{array}\right) \end{array}$$

Here, ${\vec{x}}_{\alpha }$ is the center position of the box related to state α. There exist four possible transitions since state α has two neighboring states in each direction. The rate $w_{\alpha^{-},\alpha }^{\left(r_{1}\right)}$ is the net rate of transitions into state α from the adjacent state α^−^ (i.e., the state with smaller *r*_1_), while $w_{\alpha ,\alpha^{+}}^{\left(r_{1}\right)}$ represents the rate of transitions from α to α^+^ (the state with larger *r*_1_). Similarly, $w_{\alpha^{-},\alpha }^{\left(r_{2}\right)}$ and $w_{\alpha ,\alpha^{+}}^{\left(r_{2}\right)}$ denote corresponding transitions rates between boxes arranged along the *r*_2_ direction, respectively. Of note, each rate has a sign. For example, $w_{\alpha^{-},\alpha }^{\left(r_{1}\right)}<0$ means there are more transitions from α to α^−^ than the reverse direction per unit time.

These rates can be estimated by the temporal trajectories of *r*_1_ and *r*_2_:


(7) $$\begin{array}{l} w_{\alpha ,\beta }^{\left(r_{i}\right)}=\frac{N_{\alpha ,\beta }^{\left(r_{i}\right)}-N_{\beta ,\alpha }^{\left(r_{i}\right)}}{t_{total}}. \end{array}$$

where *t*_*total*_ is the total simulation time and $N_{\alpha ,\beta }^{\left(r_{i}\right)}$ ($N_{\beta ,\alpha }^{\left(r_{i}\right)}$) is the number of transitions from state α (β) to state β (α) along the direction *r*_*i*_. For the cases where the trajectories go from one box to a non-adjacent box in a single time-step, we perform a linear interpolation to capture all the transitions between adjacent boxes.

### 4.3. Transition Path

To quantify the transition paths between the steady-state attractor states on landscape, we firstly treat a small spherical area centered on the local minimum point as a attractor area (green and megenta dashed circles in Figures 2C, 3C), denoted by *B*_1_, ⋯ , *B*_*m*_ where *m* is the total number of attractors. The transition path from attractor *B*_*i*_ to attractor *B*_*j*_ is defined as a set of trajectory points {***X***_*t*_1__, ⋯ , ***X***_*t*_*n*__} starting from *B*_*i*_ and ending at *B*_*j*_. Since the trajectory is noisy, the start point is defined as the last point in *B*_*i*_ before leaving *B*_*i*_ and the transition is finished once the trajectory wanders into the area of attractor *B*_*j*_. Each transition path corresponds to a non-linear mapping $\varphi_{k}:\left[0,t_{end}\right]\to {\mathbb{R}}^{d}$. Suppose there are *K* transition paths from *B*_*i*_ to *B*_*j*_, then the average transition path ψ_*ij*_ is defined as


(8) $$\begin{array}{l} \psi_{ij}\left(t\right)=\frac{1}{K}\overset{K}{\underset{k=1}{\sum }}\varphi_{k}\left(t\right),\;t\in \left[0,t_{end}\right]. \end{array}$$

Of note, since the noise should be large enough to drive the transitions between attractors, the time durations of each single transition trajectory is diverse. To deal with this problem, we firstly split the trajectories evenly into equal number of points and then average these points to form the average trajectory.

## Data Availability Statement

The original contributions presented in the study are included in the article/Supplementary Material, further inquiries can be directed to the corresponding author.

## Author Contributions

CL conceived and designed the study. LY performed the research. CL and LY analyzed the results and wrote the manuscript. Both authors contributed to the article and approved the submitted version.

## Funding

CL was supported by the National Key R&D Program of China (2019YFA0709502) and the National Natural Science Foundation of China (11771098).

## Conflict of Interest

The authors declare that the research was conducted in the absence of any commercial or financial relationships that could be construed as a potential conflict of interest.

## Publisher's Note

All claims expressed in this article are solely those of the authors and do not necessarily represent those of their affiliated organizations, or those of the publisher, the editors and the reviewers. Any product that may be evaluated in this article, or claim that may be made by its manufacturer, is not guaranteed or endorsed by the publisher.
