# Supplementary material for: Quantifying the Landscape of Decision Making From Spiking Neural Networks
Source: Front Comput Neurosci. 2021 Oct 28;15:740601. doi: 10.3389/fncom.2021.740601 (PMC8581041; doi:10.3389/fncom.2021.740601)
Supplement: Supplementary file 1 [file Data_Sheet_1.pdf]

Supplementary Material

SUPPLEMENTARY FIGURES

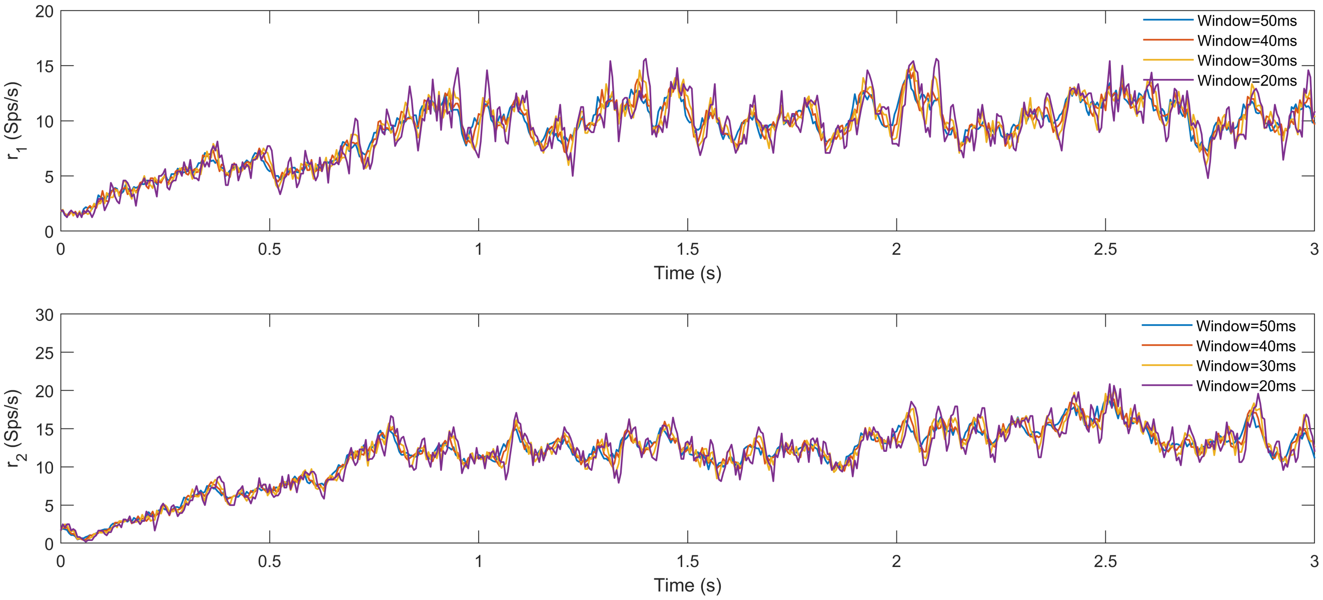

Figure S1. Trajectories of firing rate under different time windows used to calculate firing rate. With the decrease of window durations, the range of fluctuation of firing rate expands.

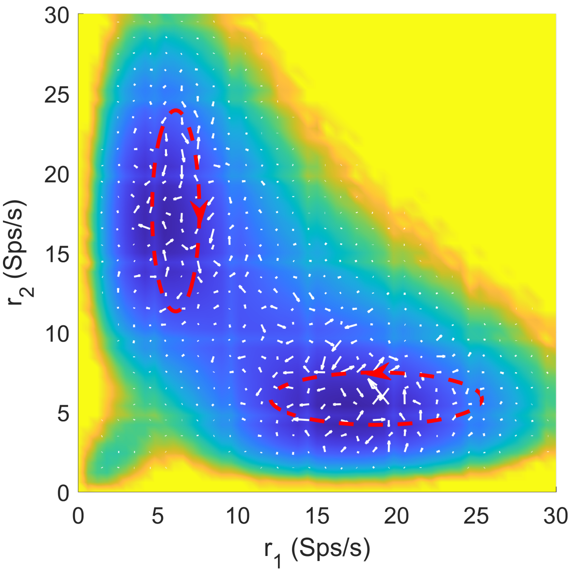

Figure S2. The landscape and probabilistic flux for the time window of $20ms$ used to calculate firing rate.

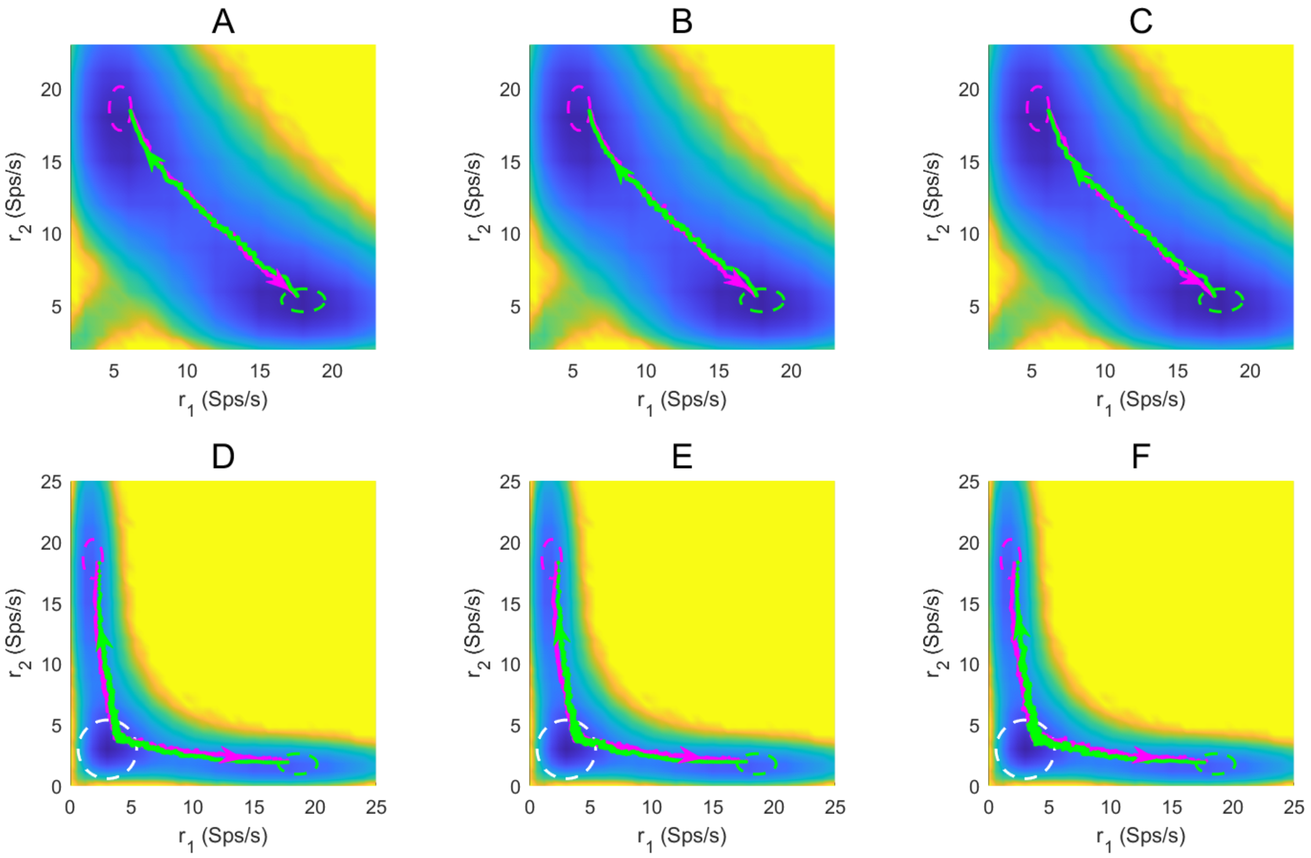

Figure S3. The transition paths using different stochastic trajectories for bistable (A-C) and tristable system (D-F).

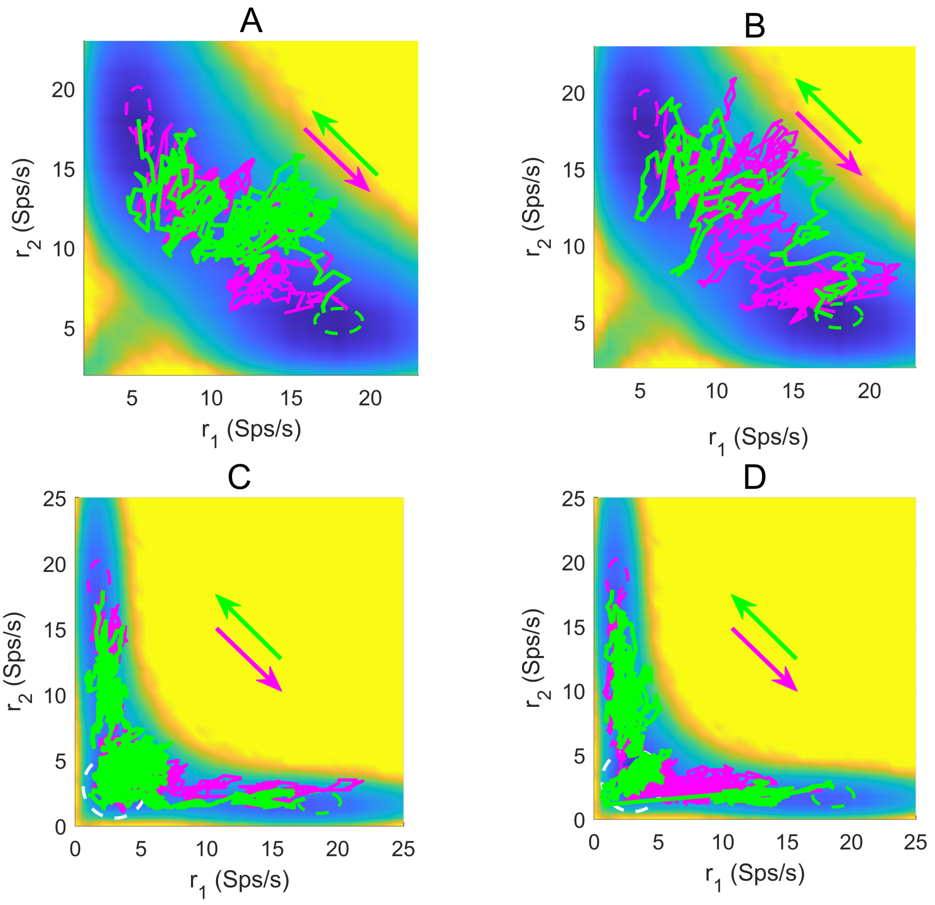

Figure S4. Single sample of transition path between attractors for bistable (A-B, corresponding to Fig. 2 in main text) and tristable (C-D, corresponding to Fig. 3 in main text) system. The green lines are single transition paths from DS1 to DS2. The megenta lines are single transition paths from DS2 to DS1.

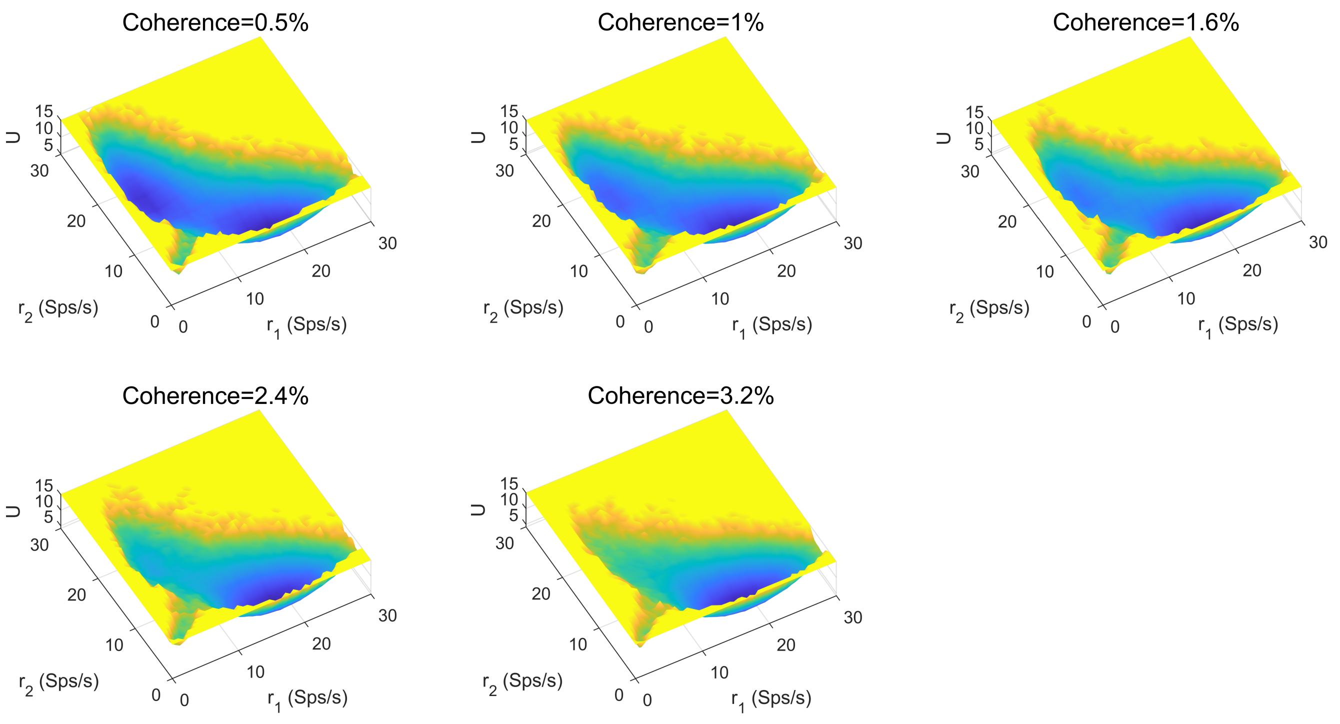

Figure S5. The asymmetric energy landscape for biased input under different motion strength corresponding to Fig. 7(A) in the main text.

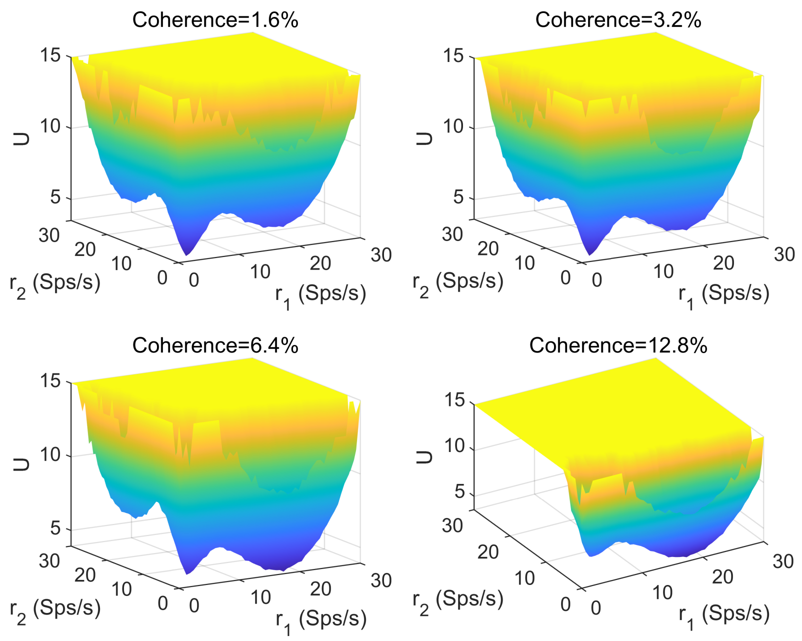

Figure S6. The asymmetric energy landscape for biased input under different motion strength corresponding to Fig. 7(C) in the main text.
